# Brain Lesion Segmentation Based on Joint Constraints of Low-Rank Representation and Sparse Representation

**DOI:** 10.1155/2019/9378014

**Published:** 2019-07-01

**Authors:** Ting Ge, Ning Mu, Tianming Zhan, Zhi Chen, Wanrong Gao, Shanxiang Mu

**Affiliations:** ^1^School of Electronic and Optical Engineering, Nanjing University of Science and Technology, No. 200, Xiaolingwei, Xuanwu District, Nanjing, Jiangsu 210094, China; ^2^School of Science, Jinling Institute of Technology, No. 99, Hongjing Avenue, Jiangning District, Nanjing, Jiangsu 211169, China; ^3^School of Information Engineering, Nanjing Audit University, No. 86, Yushan West Road, Pukou District, Nanjing, Jiangsu 211815, China; ^4^College of Computer, Nanjing University of Posts and Telecommunications, No. 9, Wenyuan Road, Yadong New District, Nanjing, Jiangsu 210023, China

## Abstract

The segmentation of brain lesions from a brain magnetic resonance (MR) image is of great significance for the clinical diagnosis and follow-up treatment. An automatic segmentation method for brain lesions is proposed based on the low-rank representation (LRR) and the sparse representation (SR) theory. The proposed method decomposes the brain image into the background part composed of brain tissue and the brain lesion part. Considering that each pixel in the brain tissue can be represented by the background dictionary, a low-rank representation that incorporates sparsity-inducing regularization term is adopted to model the part. Then, the linearized alternating direction method with adaptive penalty (LADMAP) was selected to solve the model, and the brain lesions can be obtained by the response of the residual matrix. The presented model not only reflects the global structure of the image but also preserves the local information of the pixels, thus improving the representation accuracy. The experimental results on the data of brain tumor patients and multiple sclerosis patients revealed that the proposed method is superior to several existing methods in terms of segmentation accuracy while realizing the segmentation automatically.

## 1. Introduction

**Figure 1:**
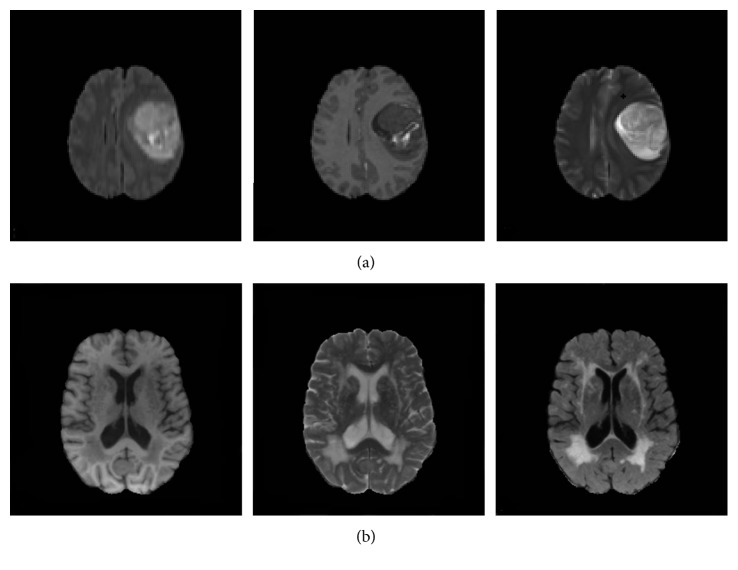
Multisequence MR images of a brain tumor patient (a) and a multiple sclerosis patient (b). (a) Multisequence images of a brain tumor, from left to right: FLAIR, T1-enhancement, and T2 sequences. (b) Multisequence images of multiple sclerosis, from left to right: T1, T2, and FLAIR sequences.

In recent years, brain diseases have become one of the most important diseases that endanger the health of human beings. The segmentation of brain lesions from brain images can be a valuable reference for the follow-up treatment of patients [1]. In the diagnosis of brain diseases, magnetic resonance (MR) imaging is the most commonly used imaging modality. Clinically, MR images of different sequences can be obtained by adjusting parameters so that brain diseases can be detected from multiple angles.  Figure 1 shows two sets of multisequence MR images. It can be seen from these images that each sequence has a different effect on the display of the brain lesion regions. As such, the complete segmentation of brain lesions according to multisequence MR images has become a research hotspot recently.

One of the most important tasks in clinical practice is to analyse multisequence MR images and segment the brain lesions to calculate the shape and volume of the lesion regions. However, having radiologists segment multiple three-dimensional (3D) images manually is time-consuming, and the segmentation results are generally not repeatable [2]. Therefore, automatic or semiautomatic segmentation methods for brain lesions are important. At present, the image segmentation methods mainly include Atlas-based methods [3–5], curve/surface evolution methods [6–8], learning-based methods [9–13], and the methods based on sparse representation (SR) [14–17] and low-rank representation (LRR) [18–21]. The results of the Atlas-based methods depend on the registration algorithm, and, to date, there is no general registration algorithm that can register the target image with the standard image accurately. Therefore, such methods are commonly used to provide geometric priors for subsequent studies. The methods based on curve/surface evolution are slow when applied to 3D image segmentation. In addition, there are so many parameters that there is no good way to balance them for different target images at this time. The learning-based methods mainly search for the optimal classifier by learning the features of samples, in which the parameters are calculated by the optimization methods without manual settings. Moreover, most of them classify the pixel points by using multidimensional features, so they are suitable for the segmentation of multisequence MR images. However, the learning-based methods only use the features of the pixels themselves, which lack spatial correlation, and the training samples often need to be labelled manually by the experts according to their clinical experience, promoting subjectivity and nonrepeatability. Although the above methods can segment the lesion regions to some extent, it is necessary to know the prior information of the lesions in advance. Therefore, they are only applicable to detect certain brain diseases. The purpose of the batch detection for brain images and the automatic segmentation of brain lesions cannot be achieved.

Because of these factors and concerns, we propose a novel automatic segmentation approach for brain lesions based on the joint constraints of LRR and SR (JCLRRSR) in this paper. Since the LRR model is able to describe the whole structure of the brain tissues in image, while the SR model is good at characterizing the local information of the pixels, the proposed method can improve the representation accuracy of the image, thereby increasing the segmentation accuracy of the brain lesions.

The rest of the paper is organized into four sections.  Section 2 introduces the SR model and the LRR model in brief.  Section 3 presents the key schemes of the proposed JCLRRSR method for segmenting the brain lesions. The experiments and discussions on the data of patients with brain tumors and multiple sclerosis are given in Section 4. Finally, the conclusions are offered in Section 5.

## 2. SR and LRR Models

### 2.1. SR Model

The SR model was derived from the requirements of signal representation, compression, and coding and was first applied only in the field of signal processing. As images have become the main expression of information, the SR model has become more widely used in the field of image processing in recent years. Here, it can not only achieve good results in classical low-level image processing problems, such as image compression, denoising, restoration, and super-resolution processing, but also perform satisfactorily regarding the problems of feature extraction, image segmentation, pattern recognition, machine learning, and some other issues. In image segmentation applications, the extracted image features of training samples are used to construct the dictionary. Then, the dictionary is used to approximate the testing sample. The class of testing sample is decided by approximate residuals in each class. At last, the classes of all testing samples generate the image segmentation results.

The basic idea of SR theory is that signals of the same class can be sparsely represented under an overcomplete dictionary [22]. The model can be expressed as follows:

(1) $$\begin{array}{c} \left\{\begin{array}{cc} \underset{A}{\min} & {\left\|A\right\|}_{0},\\ \text{s}.\text{t}. & X_{1}=DA, \end{array}\right. \end{array}$$

where *X*_1_ ∈ *R*^*N*×*M*^ is the signal matrix; *D*=[*d*_1_, *d*_2_, ⋯, *d*_*S*_] ∈ *R*^*N*×*S*^ is the dictionary matrix of the signal; *DA* is the dictionary atom; and *S* is the number of atoms in the dictionary, *S* ≫ *N*. In addition, *A*=[*a*_1_, *a*_2_, ⋯, *a*_*M*_] ∈ *R*^*S*×*M*^ is the representation coefficient matrix of the signal and denotes the zero norm of the matrix—that is, the number of nonzero elements in the matrix. Due to the nonconvexity of the zero norm, solving problem (1) is NP-hard. Considering that *A* is sparse enough, existing studies have shown that the convex relaxation method can be used for convex replacement, resulting in the following problem:

(2) $$\begin{array}{c} \left\{\begin{array}{cc} \underset{A}{\min} & {\left\|A\right\|}_{1},\\ \text{s}.\text{t}. & X_{1}=DA, \end{array}\right. \end{array}$$

where ‖·‖_1_ denotes the *l*_1_ norm of the matrix, defined as ‖*A*‖_1_=∑_*i*=1_^*S*^∑_*j*=1_^*M*^|*a*_*ij*_|, and *a*_*ij*_ is the (*i*, *j*) element of *A*.

### 2.2. LRR Model

With the development of the representation learning theory, the LRR [23, 24] has become a classic theory in the field of image processing and also has been widely used in the medical image processing and research [25–27]. It aims to search for the lowest rank representation of data under an appropriate dictionary and is good at mining data dependencies in multiple subspaces. Moreover, as compared with the subspace recovery methods based on SR, LRR is robust to noise and conducive to describing the global structure of the data, which is often unavailable in other methods. At present, the LRR model has been widely used in video patching; face recognition; and image restoration, detection, and segmentation.

For the observed signal *X* ∈ *R*^*N*×*M*^ with noise, it can be mapped to its true value signal *X*_1_ ∈ *R*^*N*×*M*^ without noise through low-dimensional subspace in high-dimensional space, where *X*_1_ is of a low-rank [23]. Let *E*=(*e*_*ij*_)_*N*×*M*_ ∈ *R*^*N*×*M*^ be the noise signal, where *e*_*ij*_ is the (*i*, *j*) element of *E*, and then *X*=*X*_1_+*E*. Since the noise signals usually account for a small part of the observed signals, the representation model of robust principal component analysis (RPCA) [28, 29] can be constructed as follows:

(3) $$\begin{array}{c} \left\{\begin{array}{cc} \underset{X_{1},E}{\min} & \text{rank}\left(X_{1}\right)+\alpha {\left\|E\right\|}_{0},\\ \text{s}.\text{t}. & X=X_{1}+E, \end{array}\right. \end{array}$$

where rank(·) denotes the rank function and *α* is a coefficient to adjust the weight of the noise term. As *X*_1_ is low-rank and *E* is sparse, the following optimization problem can be obtained by relaxing problem (3) to its convex hull:

(4) $$\begin{array}{c} \left\{\begin{array}{cc} \underset{X_{1},E}{\min} & {\left\|X_{1}\right\|}_{*}+\alpha {\left\|E\right\|}_{1},\\ \text{s}.\text{t}. & X=X_{1}+E, \end{array}\right. \end{array}$$

where ‖·‖_*∗*_ denotes the kernel norm of the matrix.

However, RPCA assumes that the data are in a single low-rank subspace, which is not suitable for the cases in which the data are in multiple subspaces. Subsequent development led to the formation of LRR theory, and the relevant model is as follows:

(5) $$\begin{array}{c} \left\{\begin{array}{cc} \underset{A,E}{\min} & {\left\|A\right\|}_{*}+\alpha {\left\|E\right\|}_{2,1},\\ \text{s}.\text{t}. & X=DA+E, \end{array}\right. \end{array}$$

where ‖·‖_2,1_ denotes the *l*_2,1_ norm of the matrix, defined as

(6) $$\begin{array}{c} {\left\|E\right\|}_{2,1}=\overset{M}{\underset{j=1}{\sum }}\sqrt{\overset{N}{\underset{i=1}{\sum }}{\left(e_{ij}\right)}^{2}.} \end{array}$$

## 3. Brain Lesion Segmentation Based on JCLRRSR

### 3.1. Data Preprocessing

We first registered the multisequence brain MR images by the registration method in the MIPAV software and corrected the grey level of the images by the method of N4ITK. The purpose of these operations was to remove the skull of the T1 sequence image and then use the remaining part as a template to remove the skull of other sequence images. After that, we adjusted the greyscale range of the images to [0, 255] by means of the following equation:

(7) $$\begin{array}{c} X^{T_{1}}=255\times \frac{I^{T_{1}}-\min\left(I^{T_{1}}\right)}{\max\left(I^{T_{1}}\right)-\min\left(I^{T_{1}}\right)}, \end{array}$$

where *I*^*T*_1_^ denotes the original image of *T*_1_ sequence and *X*^*T*_1_^ denotes the corresponding image after preprocessing. A similar preprocessing is applied on the images of other sequences.

### 3.2. Background Dictionary Construction

In order to segment the whole brain lesion regions, we regarded all of the brain tissues as background and treated brain lesions as abnormalities in the background distribution, respectively. Therefore, the background dictionary plays an important part in the proposed method and directly affects the subsequent segmentation performance of the brain lesions. In general, the background dictionary needs to meet three requirements, as follows: first, only the pixels of the brain tissue feature should be selected as the atoms, while the pixels in the lesion regions cannot be chosen; second, all categories of the brain tissue in the image should be included; and third, the number of atoms in the dictionary should be sufficient. Considering that the greyscale distribution of healthy brain tissue is relatively simple, the preprocessed normal human brain images were first classified and a certain number of pixels were extracted from the white matter, grey matter, and cerebrospinal fluid, respectively, as training samples. Then, the neighborhood of each training sample in each sequence image (the size of the neighborhood was *ω* × *ω*) was selected and converted into a greyscale vector (the length of the vector was *ω*^2^). The greyscale vectors of *k* sequences were combined to form the feature vector of each training sample (the length of the feature vector was *kω*^2^). At last, all feature vectors were combined to construct the background dictionary matrix required for the proposed method (the number of training samples was *S*, and the size of the dictionary was *kω*^2^ × *S*).

### 3.3. JCLRRSR Model

Each pixel in the brain image may correspond to one kind of brain tissue or the mixture of several kinds of brain tissue. The greyscale features of each kind of tissue can be expressed in a certain subspace, and the greyscale features of all pixels in the image should be considered in multiple subspaces. Meanwhile, the brain lesions are regarded as an abnormal form within the normal brain tissue background, which exists independently outside of all subspaces. Thus, only the pixels belonging to normal brain tissue can be represented by the background dictionary, while the pixels in lesion regions cannot be. Because of this, if we let *Y* be the high-dimensional feature matrix of the brain image to be measured, *Y* can be divided into two parts according to the LRR model (5)—that is, the background part composed of brain tissue and the brain lesion part. In model (5), *D* is the background dictionary matrix, *A* is the representation coefficient matrix, and *E* corresponds to the brain lesions.

The LRR model can effectively characterize the overall structural of the image, while the SR model is good at maintaining the local features of the pixels. Because of the different advantages of these two models, we introduced a sparse constraint for matrix *A* to the LRR model in this paper, and a new representation model for brain lesions was proposed as follows:

(8) $$\begin{array}{c} \left\{\begin{array}{cc} \underset{A,E}{\min} & {\left\|A\right\|}_{*}+\beta {\left\|A\right\|}_{1}+\alpha {\left\|E\right\|}_{2,1},\\ \text{s}.\text{t}. & Y=DA+E, \end{array}\right. \end{array}$$

where *α* and *β* are the coefficients to adjust the weight of the brain abnormalities and the sparse term, respectively.

By solving model (8) and obtaining the optimal solutions *A*^*∗*^ and *E*^*∗*^, corresponding to *A* and *E*, respectively, the response value of the *j*th pixel in *Y* belonging to the abnormal regions can be defined as follows:

(9) $$\begin{array}{c} T\left(x_{j}\right)={\left\|e_{j}^{*}\right\|}_{2}=\sqrt{\overset{N}{\underset{i=1}{\sum }}{\left(e_{ij}^{*}\right)}^{2}}, \end{array}$$

where *e*_*j*_^*∗*^ and *e*_*ij*_^*∗*^ are the *j*th column and the (*i*, *j*) element of *E*^*∗*^, respectively. If *T*(*x*_*j*_) is greater than a predetermined threshold, *x*_*j*_ can be determined as a pixel within the lesion regions.

### 3.4. Model Solving

Since the alternating direction method requires two auxiliary variables and asks for a complex matrix inverse operation in each iteration, we choose the linearized alternating direction method with adaptive penalty (LADMAP) [18, 30] to solve problem (8).

To make the objective function in problem (8) separable, we introduced an auxiliary variable *U* which satisfies *U*=*A*; then, we can replace the second term ‖*A*‖_1_ in the objective function with ‖*U*‖_1_. After that, problem (8) can be converted to the following problem:

(10) $$\begin{array}{c} \left\{\begin{array}{cc} \underset{A,E}{\min} & {\left\|A\right\|}_{*}+\beta {\left\|U\right\|}_{1}+\alpha {\left\|E\right\|}_{2,1},\\ \text{s}.\text{t}. & Y=DA+E,A=U. \end{array}\right. \end{array}$$

The Lagrange equation is as follows:

(11) $$\begin{array}{c} L\left(A,U,E,Z_{1},Z_{2},\gamma \right)={\left\|A\right\|}_{*}+\beta {\left\|U\right\|}_{1}+\alpha {\left\|E\right\|}_{2,1}+\langle Z_{1},Y-DA-E\rangle +\langle Z_{2},A-U\rangle +\frac{\gamma }{2}\left({\left\|Y-DA-E\right\|}_{F}^{2}+{\left\|A-U\right\|}_{F}^{2}\right)={\left\|A\right\|}_{*}+\beta {\left\|U\right\|}_{1}+\alpha {\left\|E\right\|}_{2,1}+f\left(A,U,E,Z_{1},Z_{2},\gamma \right)-\frac{1}{2\gamma }\left({\left\|Z_{1}\right\|}_{F}^{2}+{\left\|Z_{2}\right\|}_{F}^{2}\right), \end{array}$$

where *Z*_1_ and *Z*_2_ are the Lagrange multipliers, *γ* > 0 is the penalty parameter, and

(12) $$\begin{array}{c} f\left(A,U,E,Z_{1},Z_{2},\gamma \right)=\frac{\gamma }{2}\left({\left\|Y-DA-E+\frac{Z_{1}}{\gamma }\right\|}_{F}^{2}+{\left\|A-U+\frac{Z_{2}}{\gamma }\right\|}_{F}^{2}\right). \end{array}$$

The above multivariable optimization problem can be solved by alternately updating one variable while fixing the remaining variables. In the *k*th iteration, problem (10) can be divided into the following three subproblems:

(1) Fix *U* and *E* and update *A*, and the objective function becomes
  (13) $$\begin{array}{c} A_{k+1}=\underset{A}{\arg\min}{\left\|A\right\|}_{*}+\langle \nabla_{Af}\left(A_{k},U_{k},E_{k},Z_{1,k},Z_{2,k},\gamma_{k}\right),A-A_{k}\rangle +\frac{\theta \gamma_{k}}{2}{\left\|A-A_{k}\right\|}_{F}^{2},\\ =\underset{A}{\arg\min}{\left\|A\right\|}_{*}+\frac{\theta \gamma_{k}}{2}{\left\|A-A_{k}+\frac{\left[-D^{T}\left(Y-DA_{k}-E_{k}+Z_{1,k}/\gamma_{k}\right)+\left(A_{k}-U_{k}+Z_{2,k}/\gamma_{k}\right)\right]}{\theta }\right\|}_{F}^{2}, \end{array}$$
  where the quadratic term *f* is replaced by a first-order approximation in the *k*th step plus a neighboring operator [30, 31], ∇_*Af*_ is the derivative of *f* to *A*, and *θ*=‖*D*‖_2_^2^.
(2) Fix *A* and *E* and update *U*, and the objective function becomes
  (14) $$\begin{array}{c} U_{k+1}=\underset{U}{\arg\min\beta }{\left\|U\right\|}_{1}+\frac{\gamma_{k}}{2}{\left\|\frac{A_{k+1}-U+Z_{2,k}}{\gamma_{k}}\right\|}_{F}^{2}. \end{array}$$
(3) Fix *A* and *U* and update *E*, and the objective function becomes
  (15) $$\begin{array}{c} E_{k+1}=\underset{E}{\arg\min\alpha }{\left\|E\right\|}_{2,1}+\frac{\gamma_{k}}{2}{\left\|\frac{Y-DA_{k+1}-E+Z_{1,k}}{\gamma_{k}}\right\|}_{F}^{2}. \end{array}$$

The steps of LADMAP are shown in Algorithm 1 where the order of **step 2**, **step 3**, and **step 4** can be exchanged and Θ and Ψ are the singular value contraction operator [32] and the soft threshold contraction operator [33], respectively. Θ is defined as follows:

(16) $$\begin{array}{c} \Theta_{\lambda }\left(Y\right)=PD_{\lambda }\left(\sum \right)Q^{T}, \end{array}$$

where *D*_*λ*_(∑)=diag({max(*σ*_*i*_ − *λ*, 0)}) and *P*, ∑, and *Q* are the matrices obtained by SVD of *Y*; that is, *Y*=*P*∑*Q*^*T*^.

Ψ is defined as follows:

(17) $$\begin{array}{c} \Psi_{\varepsilon }\left[x\right]=\left\{\begin{array}{cc} x-\varepsilon , & x>\varepsilon ,\\ x+\varepsilon , & x<-\varepsilon ,\\ 0, & \text{otherwise,} \end{array}\right. \end{array}$$

where *x* ∈ *R* and *ε* > 0. When operating on a matrix or a vector, Ψ means to operate on the elements in the matrix or vector, respectively.

According to Yang et al. [34], **step 4** can be solved as follows: let *Q*_*k*_=*Y* − *DA*_*k*+1_+*Z*_1,*k*_/*γ*_*k*_, and then the *j*th column of the optimal solution *E*_*k*+1_ is

(18) $$\begin{array}{c} {\left[E_{k+1}\right]}_{j}=\left\{\begin{array}{cc} \frac{\left\|{\left[Q_{k}\right]}_{j}\right\|-\left(\alpha /\gamma_{k}\right)}{\left\|{\left[Q_{k}\right]}_{j}\right\|}{\left[Q_{k}\right]}_{j}, & \frac{\alpha }{\gamma_{k}}<\left\|{\left[Q_{k}\right]}_{j}\right\|,\\ 0, & \text{otherwise,} \end{array}\right. \end{array}$$

where [*E*_*k*+1_]_*j*_ and [*Q*_*k*_]_*j*_ are the *j*th column of the matrices.

In summary, the general algorithm of the proposed method in this paper is given in Algorithm 2.

## 4. Experiments and Discussions

### 4.1. Experimental Data

To evaluate the effectiveness of the JCLRRSR, we performed experiments on the data of two groups of patients with brain diseases. The first dataset is the multisequence MR images of patients with brain tumors, provided by the MICCAI 2012 Brain Tumor Segmentation Challenge (BraTS 2012). There are 25 patients' MR data, and each patient's data include four sequences of MR images, which are T1, T2, FLAIR, and T1-enhancement, respectively, as well as the real-world results of brain tumor regions and edema regions. The image size is 240 × 240 × 155 and the resolution is 1 × 1 × 1 mm. In the experimental comparison in this section, both tumor and edema were regarded as brain lesions. The second dataset is the multisequence MR images of patients with multiple sclerosis, provided by the ACCORD-MIND database. There are 50 patients' MR data, and each patient's data include four sequences of MR images, specifically T1, T2, PD, and FLAIR, as well as the lesion regions labelled by radiologists manually. The image size is 256 × 256 × 46 and the resolution is 0.95 × 0.95 × 3 mm. Due to the influence of the image preprocessing effect, the JCLRRSR will segment the brain lesions as well as the skull which has not been removed completely. To this end, we postprocessed the segmentation results, in which we only retained the part belonging to the brain tissue and removed the others. In the analysis of the experimental results, the Dice Score indicator was adopted to verify the accuracy of the segmentation.

### 4.2. Number of Training Samples

**Figure 2:**
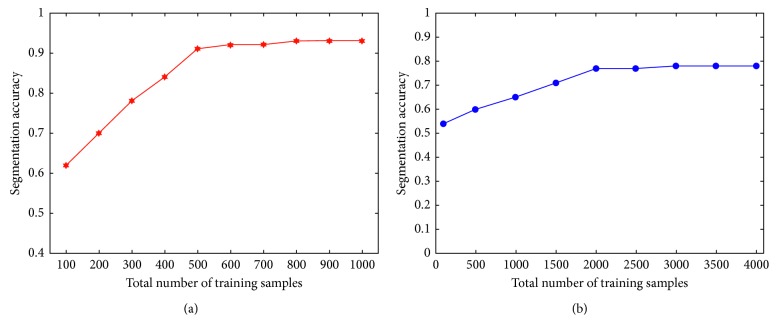
Correlation diagram between the segmentation accuracy and the total number of training samples. (a) Brain tumor segmentation. (b) Multiple sclerosis injury region segmentation.

The number of training samples in the dictionary is a key factor in the JCLRRSR. More training samples means there are more normal brain tissue samples and so the segmentation results will be better, but the computational efficiency will be lower. Conversely, the fewer the training samples, the higher the model computational efficiency but the lower the segmentation accuracy. Therefore, a clear trade-off between segmentation accuracy and computational efficiency exists. Because only the samples belonging to normal brain tissue, such as white matter, grey matter, and cerebrospinal fluid, are needed in the background dictionary and the greyscale characteristics of the three types of brain tissue are relatively close, the segmentation accuracy will reach a stable state when the training sample capacity reaches a certain number. It can be seen from the brain image that the area of white matter is larger than those of grey matter and cerebrospinal fluid. Therefore, the number of training samples we selected from the white matter was three times the number of selections made from the other two tissues.  Figure 2 shows the relationship between the total number of training samples and the segmentation accuracy of brain tumor and multiple sclerosis lesions. It can be seen from the figure that, with the increase in the number of training samples, the segmentation accuracy also increases and that when the number increases to a certain extent, the segmentation accuracy reaches a stable state. In addition, the total number of training samples needed in brain tumor segmentation is less than that in multiple sclerosis injury region segmentation, which is mainly due to the fact that the brain tumor occupies a much larger area than the multiple sclerosis injury regions. In order to balance the segmentation efficiency, the total number of training samples was set to 500 when segmenting the brain tumor and 2000 when segmenting multiple sclerosis lesions in the experiment.

### 4.3. Size of Neighborhood

**Figure 3:**
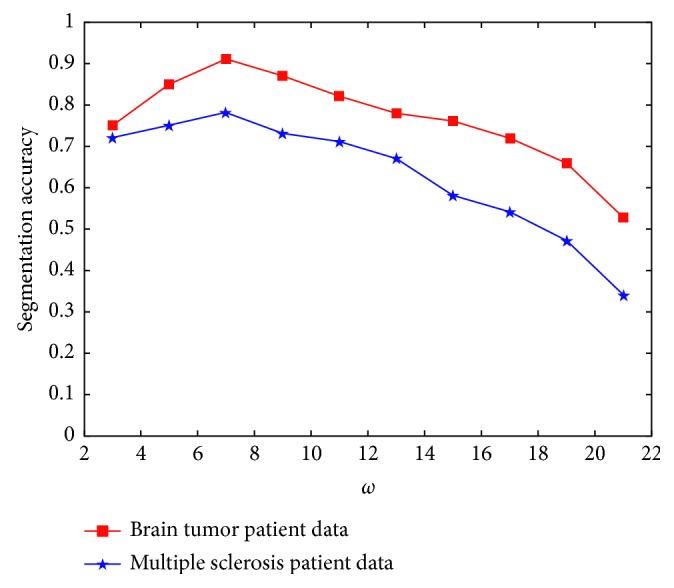
Correlation diagram between the segmentation accuracy and the size of the neighborhood.

When constructing high-dimensional features of pixels, we transformed the neighborhoods of each pixel into a vector and then merged the vectors from the different sequence images. When the image block is too large, the categories included will be inconsistent and the extracted features cannot represent the current pixel well. Conversely, when the image block is too small, the features are less and the discrimination between different pixels will not be enough. Therefore, the size of image block is another key factor in the JCLRRSR.  Figure 3 shows the effect of the neighborhood size on the segmentation accuracy of the brain tumor and the multiple sclerosis lesions. It can be seen from the figure that when the neighborhood size is set to 7 × 7, the segmentation accuracy of both the brain tumor and the multiple sclerosis lesions is optimal. The reason for this may be that the grey matter and the cerebrospinal fluid both present an elongated structure in the brain image. When the image block is too large, the central pixel and other pixels in the image block will belong to different brain tissue types. This will affect the accuracy of feature extraction and then affect the final segmentation.

### 4.4. Parameter Settings

**Figure 4:**
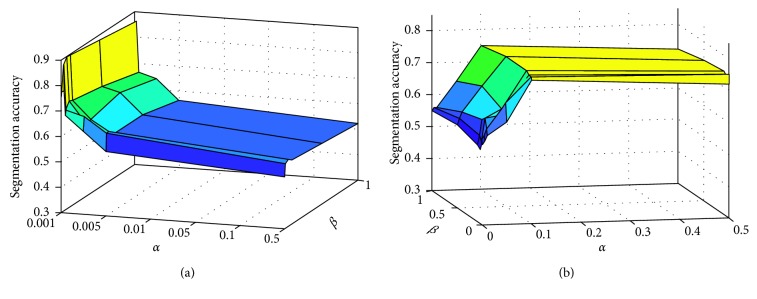
Correlation diagram between the segmentation accuracy and parameters *α* and *β*. (a) Brain tumor segmentation. (b) Multiple sclerosis injury region segmentation.

There are two parameters, *α* and *β*, involved in the JCLRRSR.  Figure 4 shows the effects of *α* and *β* on the segmentation accuracy of the brain tumor and the multiple sclerosis injury regions, where *α* takes the value in {0.001, 0.005, 0.01, 0.05, 0.1, 0.5} and *β* takes the value in {0.001, 0.01, 0.05, 0.1, 0.5, 1}. As can be seen from the figure, the algorithm is greatly affected by *α* both for brain tumor data and multiple sclerosis data. This is mainly because, although the brain tumor area is much larger than the multiple sclerosis injury regions, it contains multiple subclasses such as tumor and edema, and there are differences in the characteristics of the pixels in these regions. In this experiment, we established *α*=0.005 and *β*=0.05 for brain tumor data and *α*=0.1 and *β*=0.05 for multiple sclerosis data, respectively.

### 4.5. Lesion Segmentation Results

**Figure 5:**
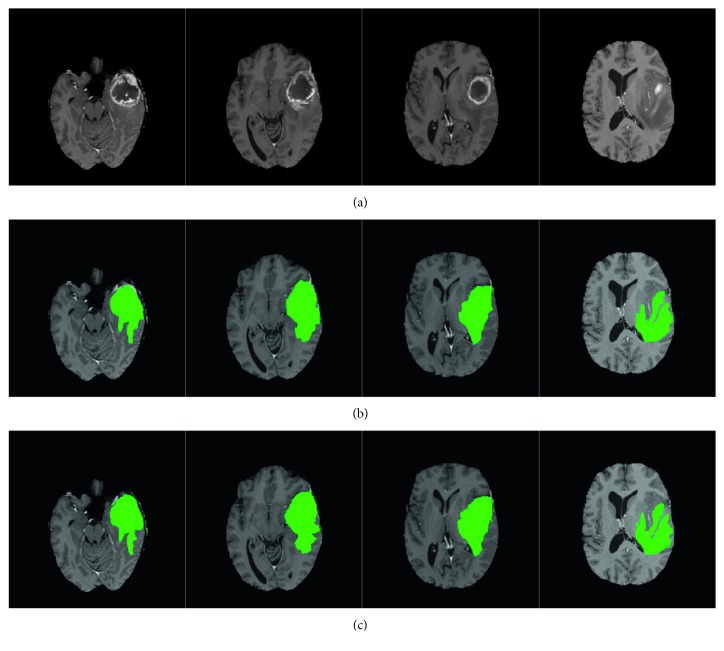
Segmentation of the brain tumor images. (a) The original brain tumor images. (b) The segmentation results obtained by the JCLRRSR. (c) The real-world results provided by the MICCAI 2012.

**Figure 6:**
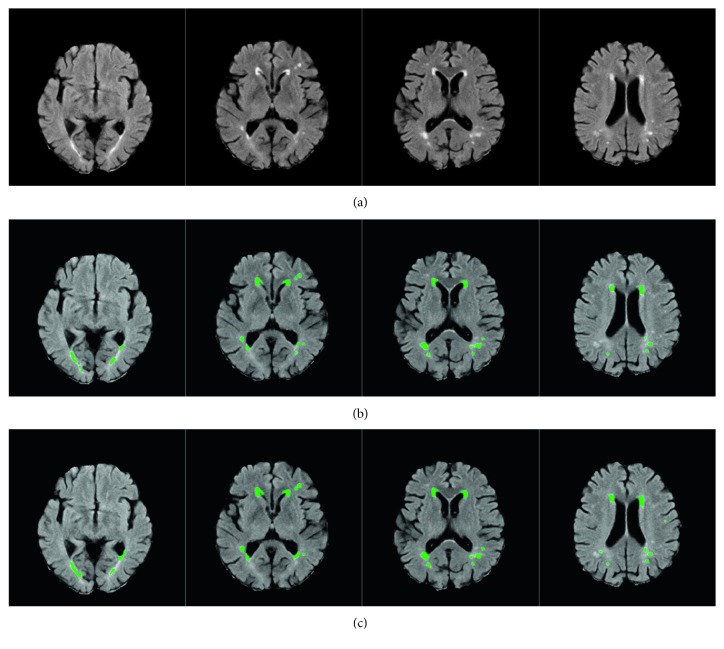
Segmentation of the multiple sclerosis images. (a) The original multiple sclerosis images. (b) The segmentation results obtained by the JCLRRSR. (c) The real-world results provided by the ACCORD-MIND.

Figures 5 and 6 show the segmentation results of the brain tumor and the multiple sclerosis injury regions, respectively. In the segmentation of the brain tumor, since the lesion regions in the image include the brain tumor and the edema around it, the JCLRRSR would detect them as a whole. If a subsequent quantitative analysis of the brain tumor is required, the test results will be further processed. The figures show that the segmentation results obtained by the JCLRRSR are close to the real-world results, which therefore meet the clinical needs. For better comparative analysis, different data subjects and different numbers of training samples are used to test several segmentation algorithms. In the brain tumor dataset, the samples are divided into high-grade and low-grade gliomas according to the degree of tumor malignancy. Separately, in the multiple sclerosis dataset, the samples are divided into big multiple sclerosis and small multiple sclerosis lesions according to the size of lesions. From Table 1, we can see that the average accuracies of SRD, LRR, and the proposed JCLRRSR method executed on different datasets and subjects have a strong correlation with the number of training samples, but the Global-RX method is not sensitive to the number of training samples. In general, these methods achieve better accuracy on HGG and BMSL because of the large targets present for these two subjects. Beside these, the JCLRRSR method can achieve optimal segmentation accuracy with different datasets and different subjects. This comparison demonstrates the superiority of the proposed method on multisequence MR images.

## 5. Conclusions

This paper presents an improved segmentation method for brain lesions. The multisequence MR images were first fused to form a high-dimensional feature matrix, during which time the neighborhood information was incorporated into the high-dimensional features of each pixel. Then, according to the proposed JCLRRSR model, the image feature matrix was decomposed and modeled under the joint constraints of LRR and SR. The model not only reflected the global structure of the image but also maintained the local information of the pixels, thus improving the decomposition accuracy. Finally, considering the computational efficiency, the LADMAP was selected to solve the model and then the brain lesions were segmented. The setting of neighborhood size, the number of training samples, and the values of parameters *α* and *β* involved in the model were discussed in detail in Section 4. In order to verify the effectiveness of the JCLRRSR approach, experiments were carried out involving the brain tumor data and the multiple sclerosis data. The experimental results revealed that JCLRRSR can not only segment brain lesions automatically but also have certain advantages in terms of segmentation accuracy as compared with other existing methods.

## Figures and Tables

**Algorithm 1:**
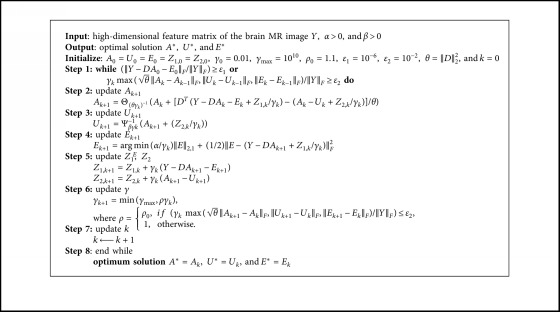
LADMAP steps.

**Algorithm 2:**
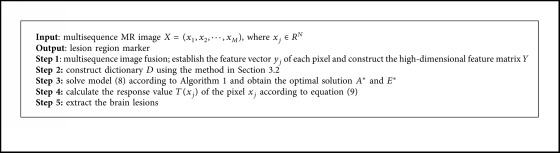
Brain lesion segmentation based on JCLRRSR.

**Table 1:** Comparison of segmentation accuracy obtained by different methods for two cases.

Lesions	Subjects	Number of training samples	Global-RX [35]	SRD [36]	LRR	JCLRRSR
Brain tumor	HGG	200	0.7542	0.6803	0.7016	0.7624
		500	0.7634	0.8153	0.8565	0.9175
		800	0.7689	0.8209	0.8602	0.9213
	LGG	200	0.7227	0.5422	0.6014	0.6624
		500	0.7272	0.7903	0.8325	0.8951
		800	0.7305	0.8023	0.8412	0.9031
	Total	200	0.7384	0.6112	0.6515	0.7148
		500	0.7503	0.8028	0.8445	0.9063
		800	0.7497	0.8116	0.8507	0.9122

Multiple sclerosis	BMSL	1000	0.6674	0.5213	0.5641	0.6425
		2000	0.6846	0.7235	0.7637	0.8026
		3000	0.6855	0.7321	0.7732	0.8242
	SMSL	1000	0.5326	0.4865	0.5245	0.6057
		2000	0.5578	0.6395	0.6835	0.7344
		3000	0.5587	0.6400	0.6910	0.7356
	Total	1000	0.6000	0.5039	0.5443	0.6241
		2000	0.6212	0.6815	0.7236	0.7685
		3000	0.6221	0.6860	0.7321	0.7799

## Data Availability

The two sets of data used to support the findings of this study are both from open datasets. Among that, one is from the MICCAI BraTS Challenge 2012 (http://www2.imm.dtu.dk/projects/BRATS2012/data.html). The other is from the ACCORDION MIND database (https://clinicaltrials.gov/ct2/show/NCT00182910).
